# Psychophysiological Reactions of Internet Users Exposed to Fluoride Information and Disinformation: Protocol for a Randomized Controlled Trial

**DOI:** 10.2196/39133

**Published:** 2022-06-16

**Authors:** Matheus Lotto, Olivia Santana Jorge, Tamires Sá Menezes, Ana Maria Ramalho, Thais Marchini Oliveira, Fernando Bevilacqua, Thiago Cruvinel

**Affiliations:** 1 Department of Pediatric Dentistry, Orthodontics and Public Health Bauru School of Dentistry University of São Paulo Bauru Brazil; 2 Department of Computer Science Federal University of Fronteira Sul Chapecó Brazil

**Keywords:** fluoride, disinformation, randomized controlled trial, social media, internet

## Abstract

**Background:**

False messages on the internet continually propagate possible adverse effects of fluoridated oral care products and water, despite their essential role in preventing and controlling dental caries.

**Objective:**

This study aims to evaluate the patterns of psychophysiological reactions of adults after the consumption of internet-based fluoride-related information and disinformation.

**Methods:**

A 2-armed, single-blinded, parallel, and randomized controlled trial will be conducted with 58 parents or caregivers of children who attend the Clinics of Pediatric Dentistry at the Bauru School of Dentistry, considering an attrition of 10% and a significance level of 5%. The participants will be randomized into test and intervention groups, being respectively exposed to fluoride-related information and disinformation presented on a computer with simultaneous monitoring of their psychophysiological reactions, including analysis of their heart rates (HRs) and 7 facial features (mouth outer, mouth corner, eye area, eyebrow activity, face area, face motion, and facial center of mass). Then, participants will respond to questions about the utility and truthfulness of content, their emotional state after the experiment, eHealth literacy, oral health knowledge, and socioeconomic characteristics. The Shapiro-Wilk and Levene tests will be used to determine the normality and homogeneity of the data, which could lead to further statistical analyses for elucidating significant differences between groups, using parametric (Student *t* test) or nonparametric (Mann-Whitney *U* test) analyses. Moreover, multiple logistic regression models will be developed to evaluate the association of distinct variables with the psychophysiological aspects. Only factors with significant Wald statistics in the simple analysis will be included in the multiple models (*P*<.2). Furthermore, receiver operating characteristic curve analysis will be performed to determine the accuracy of the remote HR with respect to the measured HR. For all analyses, *P*<.05 will be considered significant.

**Results:**

From June 2022, parents and caregivers who frequent the Clinics of Pediatric Dentistry at the Bauru School of Dentistry will be invited to participate in the study and will be randomized into 1 of the 2 groups (control or intervention). Data collection is expected to be completed in December 2023. Subsequently, the authors will analyze the data and publish the findings of the clinical trial by June 2024.

**Conclusions:**

This randomized controlled trial aims to elucidate differences between psychophysiological patterns of adults exposed to true or false oral health content. This evidence may support the development of further studies and digital strategies, such as neural network models to automatically detect disinformation available on the internet.

**Trial Registration:**

Brazilian Clinical Trials Registry (RBR-7q4ymr2) U1111-1263-8227; https://tinyurl.com/2kf73t3d

**International Registered Report Identifier (IRRID):**

PRR1-10.2196/39133

## Introduction

Dental caries in children is still a significant public health challenge in distinct populations, affecting 532 million infants worldwide, mainly in socially disadvantaged families [1,2]. Its contemporary management focuses on improving personal dietary and oral hygiene habits, as it is a sugar- and biofilm-driven disease resulting from consecutive dental demineralization processes [2,3]. In this context, fluoride-containing oral care products and drinking water play an important role in preventing dental demineralization and promoting remineralization [4-6]. Nevertheless, false or misleading content on the internet continually propagates the discouragement of fluoride usage because of possible adverse health effects [7], which supports the development of dental beliefs that could negatively impact parental oral health behaviors [8]. At the same time, fluoride refusal is a growing concern observed in pediatric dental offices, probably driven or reinforced by internet-based disinformation [9].

Indeed, diverse aspects such as innovative messages, information overload, and predisposed personal characteristics such as pre-existing beliefs, ideological motivations, and political polarization influence the spread of false or misleading web-based content [10-13]. Concurrently, digital users tend to interact with posts associated with their interests uncritically, reinforcing the emergence of echo chambers on social media [13-15]. On the other hand, people may also be susceptible to persuasive information slightly misaligned with their current motivations and behaviors (eg, falsehoods formulated to deceive called disinformation) [16,17]. This process of cognitive dissonance can cause different emotional reactions starting from discomfort or stress to a counterreaction depending on the threat to the individual’s freedom, a response known as psychologic reactance [16,18,19]. Interestingly, people with motivations more aligned to the values of persuasion messages exhibit less physiological arousal than less-aligned individuals [16]. Additionally, American adults likely to believe in conspiracy theories have more stressful life events and more significant perceived stress [20]. Nevertheless, the users' emotional reactions during the consumption of internet-based oral health information and disinformation remain uncertain.

Therefore, this study aims to evaluate the patterns of psychophysiological reactions of adults after the consumption of internet-based fluoride-related information and disinformation. The hypothesis (*H_1_*) for this randomized controlled trial indicates that there are differences in the psychophysiological reactions of adults exposed to oral health information and disinformation.

## Methods

### Trial Design

A 2-armed, single-blinded, parallel, and randomized controlled trial (trial registration number: U1111-1263-8227) will be conducted with 58 parents or caregivers of children who attend the Clinics of Pediatric Dentistry of the Bauru School of Dentistry, considering an attrition of 10% and a significance level of 5%. The participants will be randomized into test and intervention groups, being respectively exposed to fluoride-related information or disinformation presented on a computer, with simultaneous monitoring of their psychophysiological reactions, such as heart rate (HR) and 7 facial features (mouth outer, mouth corner, eye area, eyebrow activity, face area, face motion, and facial center of mass). Then, the participants will respond to questions about the utility and truthfulness of content, their emotional state after the experiment, eHealth literacy, oral health knowledge, and socioeconomic characteristics. The Shapiro-Wilk and Levene tests will be used to determine the normality and homogeneity of data, which could lead to further statistical analyses for elucidating significant differences between groups using parametric (Student *t* test) or nonparametric (Mann-Whitney *U* test) analyses. Moreover, multiple logistic regression models will be developed to evaluate the association of distinct variables with the psychophysiological aspects. Only factors with significant Wald statistics in the simple analysis will be included in the multiple models (*P*<.2). Furthermore, receiver operating characteristic (ROC) curve analysis will be performed to determine the accuracy of F_9_ (remote HR) concerning F_8_ (measured HR). For all analyses, *P*<.05 is considered significant.

**Figure 1:**
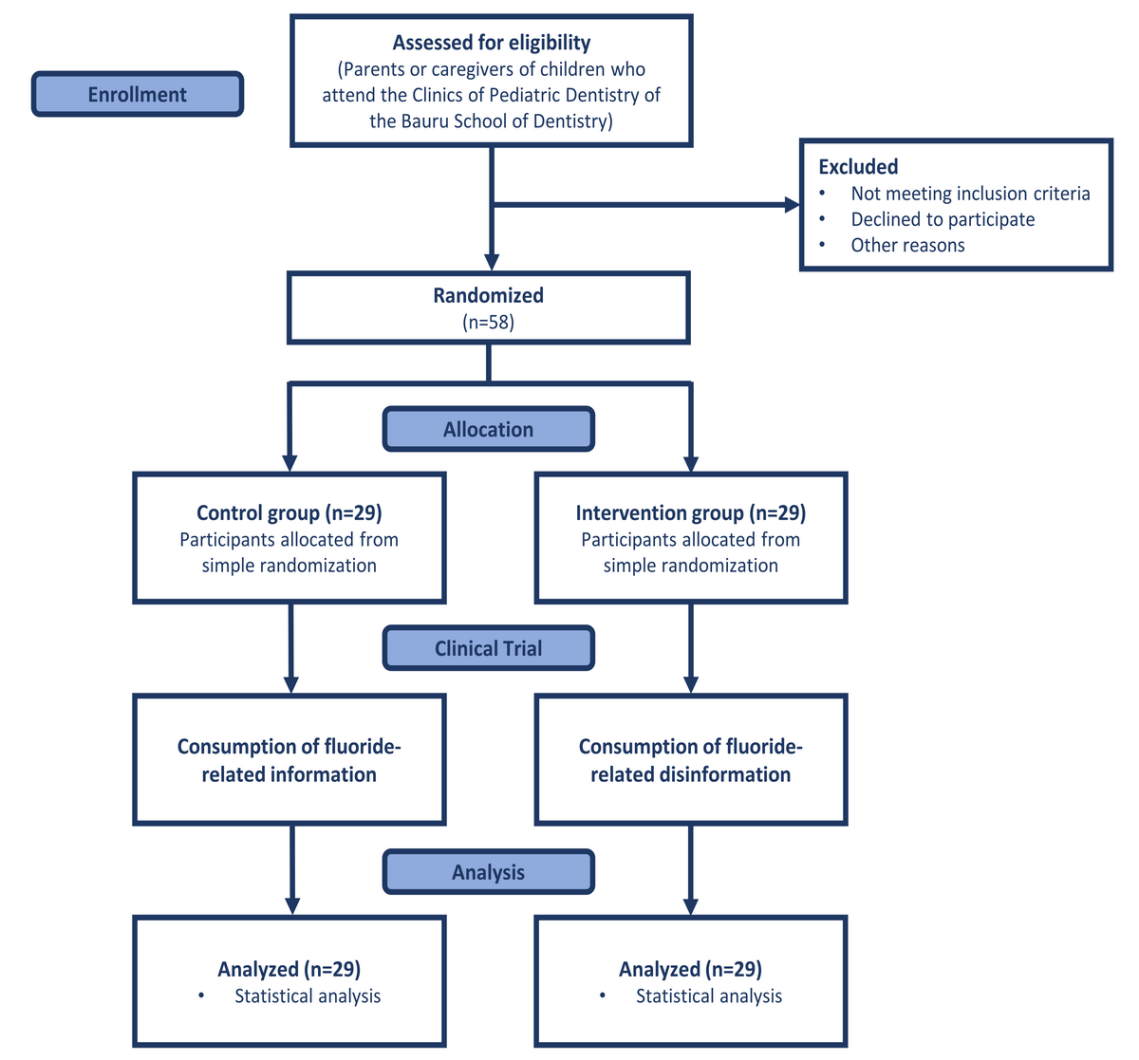
Study flowchart.

This protocol is written according to the SPIRIT (Standard Protocol Items for Clinical Trials) guidelines. Figure 1 depicts the study design synthesis [21]. The peer review reports are available in Multimedia Appendices 1 and 2.

### Participants, Intervention, and Outcomes

#### Study Setting

The study will be conducted in a reserved and silent room in the Department of Pediatric Dentistry at the Bauru School of Dentistry in the University of São Paulo, Brazil. The parents and caregivers of children who attend the Clinics of Pediatric Dentistry will be invited to participate in the study.

#### Eligibility Criteria

The participants will be recruited according to the following inclusion criteria: (1) should be parents or caregivers of children who attend the Clinics of Pediatric Dentistry of the Bauru School of Dentistry, (2) should be native Brazilians, (3) should be literates, (4) should have regular access to the internet, and (5) should agree with terms and sign the free consent form. On the other hand, people who decide to withdraw the previously signed free consent form will be excluded from the study.

#### Intervention

The experiments will be conducted based on the methodology described by Kirkwood and Minas [22]. After agreeing with the terms and signing the free consent form, the participants will be led to a silent room and will sit in front of a computer. At this moment, the researcher will instruct the participants about the test functioning (consumption of fluoride-related digital content), highlighting the presentation format and time and explaining the technical issues regarding the capture of psychophysiological reactions, besides encouraging them to persist until the end of the activity, and maintain the sitting posture during the process. To monitor the HR (1 Hz), the participants will be asked to wear 2 digital sports watches (Polar Ignite 2 and Polar Verity Sense) positioned at 7 cm away from their wrists. The HRs captured by Polar Ignite 2 will be used in the analyses, whereas the HRs captured by Polar Verity will be recorded as backup measurements.

Throughout the experiments, a camera (Canon Vixia HF R800 Full HD) will record the participants’ facial expressions. The camera will be placed on a tripod to remain slightly slanted upward and placed to face people at approximately 0.6 m. The video will be recorded in color at 60 frames per second (fps) with a resolution of 1920 × 1080 pixels and saved in the AVCHD-HD format. In addition, a spotlight will be positioned 1.6 m from participants and 45 cm above the camera level to avoid shadows on their faces during the recording. To standardize the recording of the videos, a white photographic background will be positioned approximately 60 cm behind the participants.

Initially, the psychophysiological reaction of participants will be monitored for 150 seconds while they watch a relaxing video with a classical music piece playing in the background (baseline). Next, the monitor will show 25 messages containing fluoride-related information (control) or disinformation (test) in a 6.25-minute slide presentation (15 seconds to read each message).

Each slide will show a short and straightforward message (maximum of 200 characters) for 15 seconds, which is sufficient for the participants to read and understand it [23]. The slides will be developed with a white background and contain messages in black font. By default, the messages will be aligned at the center of the slides. Additionally, the messages will be related to the main issues that emerged from a previous characterization of false or misleading fluoride-related posts on social media [24]. In this regard, we will format the original content of false or misleading posts (intervention group) to ensure a better simulation of the digital environment. Despite the difficulties in classifying distinct information disorder types, the intervention group participants will only receive disinformation because they are intended to deceive individuals intentionally [17].

At the end of the experimental session, the same video used to collect baseline data will be made available to participants for an additional 150 seconds to measure the variations in the psychophysiological parameters.

#### Questionnaires

The participants will respond to a questionnaire containing 6 questions about the self-perception of the emotional state besides the utility and truthfulness of messages, as detailed in Table 1.

Furthermore, as it is expected that age, levels of education, electronic health literacy, and oral health knowledge can influence the ability of participants in acquiring and criticizing digital content [25,26]; these outcomes will be determined through specific questionnaires.

eHealth literacy is defined as the ability of a person to search, find, understand, and evaluate digital health information and apply the acquired knowledge to address or solve a health issue [27]. It will be measured by the Brazilian version of the eHealth Literacy Scale (eHEALS) [28]. This instrument consists of 8 items linked to the consumption of health information on the internet, as demonstrated in Textbox 1. Participants must answer each question according to their self-perception using a 5-point Likert scale that has options varying from “completely agree” to “completely disagree,” with a total score ranging between 8 and 40 points [28].

The participants’ oral health knowledge will be assessed using the questionnaire proposed by Vilella et al [29], which measures knowledge related to topics of interest in pediatric dentistry, such as breastfeeding, sugar intake, bottle feeding, oral hygiene, and use of fluoride toothpaste (Textbox 2). Questions will be answered using a 3-point Likert scale, categorized as “completely agree,” “neither agree nor disagree,” and “completely disagree.” It should be noted that only the “completely agree” option is precise. Hence, the participants receive a point for each correct answer, with a final score ranging between 0 and 9.

Moreover, the participants will answer questions about their sociodemographic characteristics, self-perception of their oral and general health, and use of internet-based health information.

**Table 1:** Questionnaire about self-perception of the emotional state of participants, as well as the utility and truthfulness of messages after the consumption of fluoride-related content.

Question category		Answer options
**Emotional state**		
	1. After consuming these messages, I am feeling...	Calm
		Hectic
	2. After consuming these messages, my feeling is...	Positive
		Negative
**Utility**		
	3. Are these messages useful for your own health or for your family's health?	Yes
		No
	4. Are these messages important to other people to make their health decisions?	Yes
		No
**Truthfulness**		
	5. Have you already read or heard about these messages before this research?	Yes
		No
	6. How do you classify most of these messages?	True
		False

**Brazilian version of the eHealth Literacy Scale questionnaire presented in English.**

1. I know how to find helpful health resources on the Internet.
2. I know how to use the Internet to answer my questions about health.
3. I know what health resources are available on the Internet.
4. I know where to find helpful health resources on the Internet.
5. I know how to use the health information I find on the Internet to help me.
6. I have the skills I need to evaluate the health resources I find on the Internet.
7. I can tell high-quality health resources from low-quality health resources on the Internet.
8. I feel confident in using information from the Internet to make health decisions.

**Questionnaire about oral health knowledge of parents or caregivers.**

1. The child who is breastfeeding does not need to eat any other type of food until the six months old.
2. Up to six months old, even breastfeeding, the baby also needs to ingest water.
3. After six months old, the baby should eat foods other than maternal milk.
4. After six months old, the baby should no longer drink breast milk.
5. After one year, the baby can taste foods containing sugar.
6. The best way to give teas and juices to babies is using a bottle.
7. After six months old, the baby can be bottle-fed overnight or as much as you want.
8. While the child has baby teeth, it should wear a piece of diaper moistened with filtered water for clean teeth.
9. Babies under two years of age can use the same toothpaste as the adults.

Note: Questionnaire proposed by Vilella et al [29]

#### Outcomes

The primary outcomes will be obtained by comparing the psychophysiological reactions and self-reported emotional states of participants, as well as the self-perception of the utility and truthfulness of the content between the 2 groups.

The secondary outcomes will be related to sociodemographic characteristics, eHEALS scores, and oral health knowledge of the participants, and these are considered possible confounding factors of primary outcomes.

### Participant Recruitment Timeline

The participants will be recruited from June 2022 to December 2023. As all data will be collected simultaneously, the enrollment process will require only 1 visit from each participant.

#### Sample Size

Based on previous HR variability outcomes reported by Kirkwood and Minas [22], the sample will be composed of 58 participants, considering an effect size of 2.074 (*P*=.04) when a subject believes that an article is true, an attrition level of 10%, and a significance level of 5%.

#### Recruitment

The research team will invite the 58 parents or caregivers in person to participate in the study when they attend the Clinics of Pediatric Dentistry to their children's dental care.

#### Allocation: Sequence Generation and Concealment Mechanism

The study participants will be randomized with randomly selected block sizes using the Sealed Envelope website [30]. For this process, an operator (AMR) will generate a sequence of randomly selected block sizes to randomize participants equally into the test and intervention groups. The allocation sequence will be blinded using opaque, sealed, and consecutively numbered envelopes to ensure allocation confidentiality.

#### Implementation

A researcher not involved in the experiments (TSM) will invite the parents or caregivers to participate in the study, taking them directly to the experimental room. Before starting the intervention, the envelopes indicating the group allocated to the participants will be taken to the pretrained examiner (ML) by another operator (OSJ). Then, the allocated group will be known only to the trained examiner (ML), who will open the envelope alone immediately before starting the experiment. He will conduct the experiments, including collecting the informed consent forms and administering the questionnaires.

#### Blinding

The parents or caregivers, researchers not involved with the experiments (TSM and OSJ), and the data analysts (TC and FB) will be blinded to the participants’ allocation groups.

### Data Collection, Management, and Analysis

First, the HR data of the participants will be exported from Polar Ignite 2 and Polar Verity Sense to Microsoft Excel (Microsoft Corporation) as.csv files. Further, the sociodemographic, oral health knowledge, self-perception about information, and eHEALS data will be manually entered into Excel sheets. Moreover, the videos will be exported from the camera and stored in a closed repository for further analysis, according to the findings of Bevilacqua et al [31,32]. Notably, the features were designed based on previous reports regarding the potential to distinguish participants' emotional states [31-34]. As a result, 9 psychophysiological features will be calculated, with 7 related to facial activity and 2 to HR, as summarized in Table 2.

**Table 2:** Description of the psychophysiological characteristics to be measured in the clinical trial.

Feature number	Facial landmark	Description
F_1_	Mouth outer	Monitor the zygomatic muscle
F_2_	Mouth corner	Monitor the zygomatic muscle
F_3_	Eye area	Monitor the orbicularis oculi muscle (eg, blinking)
F_4_	Eyebrow activity	Monitor the corrugator muscle
F_5_	Face area	Monitor facial movement to and away from the camera
F_6_	Face motion	Describe the total distance the head has moved in any direction in a short period
F_7_	Facial COM^a^	Describe the overall movement of all 68 facial landmarks
F_8_	Measured HR^b^	Measurement of HR using Polar Ignite 2 and Polar Verity Sense
F_9_	Remote HR	Estimated measurement of HR using the rPPG^c^ technique

^a^COM: center of mass.

^b^HR: heart rate.

^c^rPPG: remote photoplethysmography.

Features F_1_ to F_7_ are grounded on 68 facial landmarks automatically detected using constrained local neural fields (CLNFs) and are calculated using the Euclidian distance between those facial landmarks [31,32]. However, subjects have unique facial shapes and characteristics, which could prevent their comparison. Thus, we will first calculate a normalization coefficient for the Euclidian distance between the upper and lowermost anchor landmarks to mitigate this problem [31]. Additionally, feature F_9_ is based on remote estimations of HR performed using the established remote photoplethysmography  (rPPG) technique [35]. This is an extremely resilient technique when estimating the HR under challenging conditions and could be combined with the disinformation consumption scenario.

Statistical analysis will be performed using Stata 17.0 software (StataCorp LLC, College Station, TX, USA). The questionnaire and psychophysiological data will be expressed through descriptive analysis (mean, SD, median, minimum, maximum, and percentage of variation). Moreover, the Shapiro-Wilk and Levene tests will be performed to analyze the normality and homogeneity of data to determine significant differences between groups by the Student *t* test (parametric analysis) or Mann-Whitney *U* test (nonparametric analysis). Further, multiple logistic regression models will be developed to evaluate the association of distinct variables with the psychophysiological aspects. Only factors with significant Wald statistics in the simple analysis will be included in the multiple models (*P*<.2). Furthermore, ROC curve analysis will be performed to determine the accuracy of F_9_ concerning F_8_. For all analyses, *P*<.05 will be considered statistically significant.

It is noteworthy that anonymized data will be shared in a public repository after the end of this study.

### Monitoring

#### Data Monitoring

The authors will assume responsibility for independent regulation of data collection, management, and analysis.

#### Hazards

People can feel confused about the trustworthiness of the consumed content and consequently develop negative oral health beliefs. Thus, after the intervention, the trained examiner will clarify all aspects of the information and disinformation for each individual. In this way, they will resolve the doubts and possible misunderstandings regarding fluoride consumption, whether in terms of oral hygiene or drinking water. As a result, the present study also serves as a health education measure for the participants.

#### Auditing

Data management and analysis will be conducted by 1 statistical expert from the research team (TC). If necessary, data inconsistencies will be verified, corrected, and registered.

### Ethics and Dissemination

#### Research Ethics Approval

This study was reviewed and approved by the Council on Ethics in Human Research from the Bauru School of Dentistry (CAAE: 53483821.0.0000.5417), registered in the Brazilian Clinical Trials Registry (RBR-7q4ymr2) and assigned with the universal trial number U1111-1263-8227.

#### Consent and Assent

Informed consent will be provided and assigned by the participants.

#### Confidentiality

Identification numbers will be used to ensure participant confidentiality during data analysis.

#### Availability of Data

All raw data will be available in an open repository.

#### Ancillary and Posttrial Care

This study is also intended to serve as an oral health education strategy. Thus, participants in the test group will be informed about the falsehood provided, aiming to avoid the development of negative health beliefs. Moreover, all questions about the content consumed will be clarified for all the participants. In addition, participants are expected to observe how disinformation can be harmful in managing their health.

#### Dissemination Policy

The findings will be reported in high-impact dental or medical informatics journals.

## Results

From June 2022, parents and caregivers who frequent the Clinics of Pediatric Dentistry at the Bauru School of Dentistry will be invited to participate in the study and will be randomized into 1 of 2 groups (control or intervention). Data collection is expected to be completed in December 2023. Subsequently, the authors will analyze the data and publish the findings of the clinical trial by June 2024.

## Discussion

### Principal Findings

To the best of our knowledge, it is the first study that will evaluate the differences in psychophysiological reactions of internet users exposed to or not exposed to disinformation. Given the proposed hypothesis, we expect that individuals exposed to fluoride-related disinformation will present more psychophysiological reactions during the experiments because of stressors provided by the consumption of novel content. Additionally, those who disagree with the content due to previous health beliefs tend to show more psychophysiological reactions, also considering the cognitive dissonance process [16]. Moreover, it is expected that younger adults with higher levels of eHealth literacy and oral health knowledge probably will feel less persuaded by disinformation than their older counterparts. Finally, we hope to obtain promising results pertaining to the comparison of F_9_ (remote HR) to F_8_ (measured HR), enabling remote validation and measurement of this parameter in future studies on information disorder.

### Comparison to Prior Work

Notably, the proposed analysis proved effective in detecting remote emotions in experiments with games [31], but it is still necessary to understand its strengths and limitations in the information disorder context. Nevertheless, it could be a precursor to developing artificial intelligence models to detect false health content automatically and prevent consumption in digital environments. In addition, the results can help people who are more susceptible to believe in fluoride-related false messages found on the internet better understand such information.

Furthermore, the present methodology adds a new perspective to the study of information disorder. Currently, this research field focuses on internet-based surveillance of falsehoods on social media to formulate public health measures and policies [24,36,37], besides proposing definitions and taxonomies for a better understanding of this phenomenon [17,38,39]. In this context, previous studies have detected the high prevalence of false or misleading fluoride-related content on distinct social networks [7,24,40,41]. On the other hand, investigations about disinformation concerning the user perspective are still lacking.

### Strengths

Although desirable, the mitigation of false or misleading messages from computational detection measures that screen and remove posts on social media does not seem to be enough to end the consumption and spread of digital falsehoods [42]. Indeed, the overload of information on internet channels makes data screening difficult because existing artificial intelligence–based systems cannot cover all health issues [11,43]. Considering this scenario, the proposed methodology is advantageous because it investigates the information disorder phenomenon in terms of the users' reactions, instead of only analyzing it from a content perspective. Furthermore, it is possible to validate the collection of HR data from psychophysiological facial reactions using neural network modeling, which would enable remote monitoring of users through an ordinary camera using a computer [31]. Thus, people would have an additional tool to avoid consuming disinformation.

### Limitations

The measurements of HR and psychophysiological reactions using specialized technical equipment can be uncomfortable for participants during the experiments, which would influence their stress levels. Despite this, we will use equipment that can provide greater comfort, such as a sports watch that will be attached to the wrist instead of a chest heart reader, besides providing a comfortable air-conditioned experimental room.

### Conclusions

Our randomized controlled trial aims to determine if there are identifiable differences in the psychophysiological reactions among individuals who consume true or false fluoride-related digital content. The evidence produced can support the development of further studies and digital strategies, benefiting research, businesses, and communities.

## Appendix

Multimedia Appendix 1 Peer-review report from São Paulo Research Foundation - Part 1.

Multimedia Appendix 2 Peer-review report from São Paulo Research Foundation - Part 2.

## Abbreviations

CLNF: constrained local neural field
eHEALS: eHealth Literacy Scale
HR: heart rate
ROC: receiver operating characteristic
rPPG: remote photoplethysmography
