# Supplementary material for: Psychophysiological Reactions of Internet Users Exposed to Fluoride Information and Disinformation: Protocol for a Randomized Controlled Trial
Source: JMIR Res Protoc. 2022 Jun 16;11(6):e39133. doi: 10.2196/39133 (PMC9247811; doi:10.2196/39133)
Supplement: Multimedia Appendix 1 [file resprot_v11i6e39133_app1.pdf]

VISUALIZAÇÃO DE DESPACHO

**Processo**	2019/27242-0
**Linha de Fomento**	Programas Regulares / Bolsas / No País / Doutorado - Fluxo Contínuo
**Situação**	Em Execução
**Vigência**	01/01/2021 a 30/04/2024
**Beneficiário**	Matheus Lotto de Almeida Souza
**Responsável**	Thiago Cruvinel da Silva
**Vínculo Institucional do Processo**	Faculdade de Odontologia de Bauru/FOB/USP
**Título**	Fake news em saúde: validação de um modelo de redes neurais para detecção de desinformação em Odontopediatria por meio de reações psicofisiológicas de usuários da Internet

Folha de Despacho para Proposta Inicial - Doutorado

Resultado

Denegado

Datas do Despacho

**Emitido em :**	01/04/2020

Orçamento Consolidado

Benefícios	Solicitado		Despacho	
	Valor (R$)	Valor (US$)	Valor (R$)	Valor (US$)
Custeio				
Auxílio Instalação	0,00	0,00	0,00	0,00
Despesas de Transporte	0,00	0,00	0,00	0,00
Reserva Técnica de Bolsa	37.668,24	0,00	0,00	0,00
Total	37.668,24	0,00	0,00	0,00
Bolsas				
Doutorado	125.560,80	0,00	0,00	0,00
Total	125.560,80	0,00	0,00	0,00
Total Geral	163.229,04	0,00	0,00	0,00

Dados de Execução

**Data Início**	15/06/2020
**Duração**	36 mês(es)
**Data Término**	14/06/2023
**Área de alocação de recursos**	Saúde
**Relatório Científico (Quantidade)**	1
**Relatório Científico (Datas)**	01/01/1900
**Ata de Defesa (Quantidade)**	1
**Ata de Defesa (Datas)**	01/01/1900
**Prestação de Contas (Quantidade)**	1
**Prestação de Contas (Datas)**	01/01/1900
**Permite prorrogação da Bolsa?**	Não

Observações / Transcrições / Frases

Observações ao Responsável

Comunicamos que sua solicitação de Bolsa de Doutorado, constante do processo acima referido, foi analisada pela FAPESP, e não pôde ser aprovada com base nas considerações descritas abaixo.

A demanda qualificada no Programa de Bolsas de Doutorado no País é muito superior ao número de bolsas disponíveis para concessão. Por isso, a FAPESP já não pode atender a mais que uma fração das solicitações incondicionalmente recomendadas no mérito por sua assessoria externa. Por essa razão, todas as solicitações são submetidas a um processo de Análise Colegiada altamente competitivo, no âmbito das Coordenações de Área e Adjunta. Nesse processo, define-se a posição relativa de cada solicitação numa escala de prioridades, conforme seu grau de excelência nos itens Projeto, Candidato e Orientador. Para essa definição, são considerados especialmente: o grau de originalidade, definição dos objetivos, fundamentação teórica e adequação metodológica do projeto, o grau de competência do orientador na área específica do projeto, medido por sua produtividade recente nessa área, e o potencial de pesquisa do candidato, medido por seu histórico escolar e acadêmico.
Em igualdade de condições, são atendidas prioritariamente: (a) solicitações diretamente vinculadas a projetos já apoiados pela FAPESP em suas linhas de auxílio à pesquisa - especialmente a de projetos Temáticos, projetos CEPID e projetos Jovem Pesquisador; (b) solicitações fundamentadas em projetos que tenham resultado de pesquisas já realizadas pelos candidatos com bolsas da FAPESP; (c) no caso de solicitações de bolsas de doutorado e doutorado-direto, aquelas encaminhadas por candidatos que concluíram recentemente seus cursos de mestrado ou de graduação.

A presente solicitação foi submetida à análise colegiada e não obteve o grau de prioridade necessário para seu atendimento.

Para conhecimento do conteúdo do despacho, por favor, acesse o Sistema SAGe (www.fapesp.br/sage), selecionando o item do menu Meus Processos>>Número do Processo e, em Mais Informações, a opção Despacho.

Informamos que a transcrição do parecer está disponível exclusivamente para o orientador.

Para qualquer consulta ou comunicação sobre esta correspondência, por favor, use exclusivamente os serviços do "Converse com a FAPESP" em www.fapesp.br/converse.

Permanecemos à disposição para futuras solicitações.

Atenciosamente,

Carlos Henrique de Brito Cruz
Diretor Científico

Frases para o Responsável

Não há frases associadas.

Transcrição de Parecer para o Responsável

ANÁLISE GERAL DA PROPOSTA

A Fapesp denomina "Proposta" o conjunto de três partes a serem avaliadas, composto por: 1. Projeto de Pesquisa; 2. Histórico Escolar e Acadêmico do Candidato; e, 3. Histórico de Pesquisa do Orientador. Por favor preencha este item depois de preencher o restante do formulário.

O estudo proposto apresenta um tema inovador e atual: fake news e utilização de aprendizado de máquina para desfechos em saúde.
O Projeto de Pesquisa está bem escrito e apresenta uma parte que faz parte da linha de pesquisa e estudos anteriores (avaliação de conteúdos de Odontopediatria nas mídias) e outra parte nova que propõe trabalhar com redes neurais (aprendizado de máquina).
O histórico escolar do candidato durante a graduação foi bom, com media ponderada 7,5 e sem reprovações, e no Mestrado tem sido aprovado nas disciplinas. No histórico acadêmico o candidato apresenta 9 publicações sendo 2 delas " in press", sendo primeiro autor em 3 destas publicações. Tem 1 financiamento vigente da FAPESP.
O Historico de Pesquisa do Orientador é muito bom para o tempo de dedicação exclusiva que apresenta, com publicações relevantes associadas ao tema.

Por favor, analise o PROJETO DE PESQUISA proposto, conforme roteiro abaixo:

Analise a definição, a pertinência, a originalidade dos objetivos e a importância da contribuição pretendida para a área do conhecimento em que o projeto proposto se insere.

Segundo os proponentes os resultados deste estudo permitirão aos usuários da Internet a adequada seleção de conteúdos na era da pós-verdade, caracterizada pela maximização de crenças e dificuldade no reconhecimento dos fatos pautados em evidências científicas. Para tal contam com 4 objetivos:
(1) Avaliar a tendência de buscas estruturadas dos usuários da Internet sobre temáticas ligadas à desinformação em Odontopediatria;
(2) Realizar a infovigilância dos conteúdos não-estruturados do Twitter relacionados à desinformação em Odontopediatria;
(3) Determinar as reações psicofisiológicas de mães ao consumirem desinformação relacionada à saúde bucal de crianças;

(4) Validar a capacidade de detecção remota de conteúdos de desinformação relacionada à saúde bucal de crianças pelas reações psicofisiológicas dos indivíduos.

Avalio que os 2 primeiros objetivos fazem parte de uma linha de pesquisa e publicações do Proponente e assim podem continuar contribuindo, entretanto a parte inovadora está nos objetivos 3 e 4, que poderá se tornar uma contribuição importante na medida que consiga atingir tais objetivos ao esclarecer (aprofundar) um pouco melhor como pretende atingi-los.

Analise a fundamentação científica e os métodos empregados.

A metodologia proposta não esclarece como serão considerados os diferentes níveis de alfabetização em saúde eletrônica para o estudo clinico randomizado (para atender objetivos 3 e 4). Na intervenção serão constituídos 2 grupos (informação adequada e desinformação), sendo avaliados batimentos cardíacos e nível de agitação e veracidade.

Na proposta está descrito:" Em ambos os casos, o procedimento de classificação do estado dos sujeitos será realizado posteriormente ao experimento. Ele será baseado na extração dos sinais psicofisiológicos a partir dos vídeos dos sujeitos utilizando-se visão computacional. Os sinais extraídos dos participantes, bem como as meta-informações sobre o tom emocional e veracidade de cada notícia consumida, serão utilizados para treinar uma rede neural apta a identificar dois estados, consumindo informação adequada ou desinformação."

Falta esclarecer as diferentes combinações, no sentido de experiências prévias que estas mães ja obtiveram e como irão reagir, independente da veracidade das informações. Parece um pouco mais complexo e talvez necessitasse de uma co-orientação de pessoa com experiência nestas avaliações emocionais.

Analise a adequação do projeto a um programa de doutorado e viabilidade de sua execução dentro do prazo previsto.

São 4 objetivos, sendo que para cumprir os 2 últimos será realizado um estudo clinico randomizado. Definindo melhor a metodologia, há viabilidade de execução.

Caso se trate de um candidato que já tenha iniciado o doutorado, avalie o andamento do projeto de pesquisa e a viabilidade de sua execução no prazo previsto.

Não se aplica, pois candidato ainda não finalizou Mestrado (ou não apresentou documentos relacionados a Defesa).

Conclusão sobre a análise do Projeto de Pesquisa apresentado. (Preenchimento obrigatório)

[ ] Excelente
[ ] Muito boa
[ ] Muito boa, com algumas deficiências facilmente sanáveis
[ ] Boa
[X] Boa com deficiências
[ ] Regular
[ ] Com sérias deficiências

Por favor, analise o HISTÓRICO ESCOLAR E ACADÊMICO DO CANDIDATO. As bolsas de Doutorado se destinam a estudantes com bom desempenho acadêmico avaliado, principalmente, pelo histórico escolar de graduação e pós-graduação. A concessão a estudantes com histórico escolar irregular, exibindo um padrão de reprovações ou aprovações com nota mínima, é possível apenas em circunstâncias excepcionais à vista de outras evidências sobre o potencial acadêmico do candidato, como por exemplo, projeto bem sucedido de Iniciação Científica.

Histórico Escolar do candidato.

Na graduação foi aprovado em todas disciplinas, obtendo media ponderada de 7,5 sendo classificado como 28o. de uma turma com 50 alunos.

Histórico Acadêmico do candidato (participação em projetos de pesquisa, bolsas anteriores, publicações científicas, premiações, etc.).

Apresenta 9 publicações sendo 2 delas " in press", sendo primeiro autor em 3 destas publicações.
Tem 1 financiamento vigente da FAPESP.
Desde 2015 acumula 5 menções honrosas.

Outros itens que compõem a descrição das atividades acadêmicas, científicas e profissionais desenvolvidas pelo candidato.

O beneficiário realiza pesquisas na área de eSaúde desde 2014, sob orientação do Prof. Dr. Thiago Cruvinel. Foi contemplado com três bolsas FAPESP, sendo duas de iniciação científica e uma de mestrado (em vigor) (processos nº 2014/21292-2 e 2017/25899-7).

Conclusão sobre a análise do Histórico Escolar e Acadêmico do Candidato. (Preenchimento obrigatório)

[ ] Excelente
[X] Muito bom
[ ] Bom
[ ] Regular
[ ] Com sérias deficiências

Por favor, analise o HISTÓRICO DE PESQUISA DO ORIENTADOR, seguindo o roteiro abaixo:

Qualidade e regularidade da produção científica e/ou tecnológica. Elementos importantes para essa análise são: lista de publicações em periódicos com seletiva política editorial; patentes em que figure como inventor; outros instrumentos de propriedade intelectual; resultados de pesquisa efetivamente transferidos e adotados por empresas ou pelo governo; e outras informações que possam ser relevantes.

Desde 2013 está vinculado com regime de dedicação exclusiva na disciplina de Odontopediatria da Faculdade de Odontologia de Bauru. Das 10 publicações mais relevantes, de alguma forma todas se relacionam a esta linhada pesquisa.

Experiência e competência demonstradas na liderança de projetos de pesquisas relacionados ao tema da proposta em análise

Sim, obteve financiamentos no tema, orientou alunos e tem publicações relacionadas.

Experiência internacional em pesquisa após o doutoramento ou ter demonstrado participação ativa em redes internacionais de colaboração em pesquisa.

Estagiou por 6 meses na ACTA Academisch Centrum Tandheelkunde Amsterdam -Holanda (2010).

Capacidade demonstrada para formar pesquisadores, com destaque para a atividade recente de orientação de estudantes.

Finalizou a orientação de 13 alunos de Iniciação Cientifica e 4 de Mestrado.

Resultados obtidos pelo orientador com financiamentos anteriores da Fapesp.

Obteve financiamentos anteriores com a FAPESP (anos de 2015 e 2018)

Disponibilidade para orientação considerando o regime de trabalho e o número total de estudantes sob a sua orientação.

Sim, haverá disponibilidade considerando o tempo integral e numero de alunos sob sua orientação

----

Regime de trabalho.

Integral dedicação exclusiva.

Número atual de orientandos.

3 IC
2 MS
0 DD
3 DR

Comentários

É revisor de 33 periódicos. Obteve 7 premios de menção honrosa em 2019, sendo um deles na SBPqO. Finalizou a orientação de 13 alunos de IC e 4 de M.

Outras considerações sobre a produção científica, tecnológica (patentes, transferência de tecnologia etc.) e acadêmica do orientador, relevantes para a Análise da viabilidade da proposta.

O orientador vem pesquisando sobre este tema e tem publicado sobre o assunto, aprofundando a discussão do uso e conteúdos sobre Odontopediatria nas mídias.

Conclusão sobre a Análise do Histórico de Pesquisa do Orientador. (Preenchimento obrigatório)

[ ] Excelente
[X] Muito Bom
[ ] Bom
[ ] Regular
[ ] Com sérias deficiências

DEFICIÊNCIAS NOTADAS (Se algum item estiver assinalado, as razões devem estar indicadas no quadro correspondente do formulário)

Sobre o Projeto de DR:

[ ] Projeto com objetivos mal definidos, excessivos ou incongruentes
[ ] Projeto com objetivos excessivamente limitados
[ ] Projeto pouco original
[ ] Contribuição pouco significativa para a área de conhecimento
[X] Fundamentação científica insuficiente/metodologia inadequada
[ ] Inadequado para um programa de Doutorado
[ ] Viabilidade de execução questionável
[ ] Andamento do projeto é inadequado, comprometendo a viabilidade de execução do mesmo

----

Sobre o Histórico Escolar e Acadêmico do Candidato

[ ] Histórico escolar deficiente
[ ] Candidato com potencial não evidenciado

Sobre o histórico de Pesquisa do Orientador

[ ] Produção científica ou tecnológica que não atesta significativo rendimento da atividade de pesquisa
[ ] Experiência insuficiente na área de pesquisa em que se insere o projeto, podendo comprometer a sua viabilidade
[ ] Experiência internacional em pesquisa insuficiente após o Doutoramento
[ ] Capacidade de orientação não evidenciada
[ ] A produção científica ou tecnológica resultante de auxílios anteriores é insatisfatória
----
Outras deficiências (DR):

[ ] Doutorado iniciado há mais de dois anos
[X] Outras

Outras deficiências. Justificar:

Como observação: Candidato ainda não finalizou Mestrado (teve inicio em 13/03/2019 com qualificação em 10/12/2018).

Parecer das Coordenações

Proposta não recomendada com base no parecer da assessoria sobre o projeto.

Frases para Termo de Outorga

Não há frases associadas.

Orçamento Detalhado - Quadros Resumos

Reserva Técnica de Bolsa - Solicitado

**Percentual para Reserva Técnica (País)**	30,00 %
**Percentual para Reserva Técnica (Exterior)**	0,00 %
**Dólar FAPESP**	4,30
**Valor da Reserva Técnica (R$)**	37.668,24
**Valor da Reserva Técnica (US$)**	0,00

Reserva Técnica de Bolsa - Despacho

**Percentual para Reserva Técnica (País)**	30,00 %
**Percentual para Reserva Técnica (Exterior)**	0,00 %
**Dólar FAPESP**	5,20
**Valor da Reserva Técnica (R$)**	0,00
**Valor da Reserva Técnica (US$)**	0,00

Bolsa

**Beneficiário**	Matheus Lotto de Almeida Souza	
**Modalidade**	Solicitado<br>Doutorado - DR-1	Despacho<br>Doutorado - DR-1
**Duração**	36 mês(es)	
**Data Início**	15/06/2020	15/06/2020
**Moeda**	R$	R$
**Valor Unitário**	3.010,80	3.010,80
**Valor Total das**	125.560,80 reais	0,00 reais

Mensalidades

Justificativa

Bolsas do Beneficiário

Nenhuma bolsa encontrada.

Orçamento Detalhado - Itens de despesa

Reserva Técnica de Bolsa

**Percentual para Reserva Técnica (País)**	30,00 %
**Percentual para Reserva Técnica (Exterior)**	0,00 %
**Dólar FAPESP**	5,20
**Valor da Reserva Técnica (R$)**	0,00
**Valor da Reserva Técnica (US$)**	0,00

Bolsa

**Modalidade**	Doutorado - DR-1
**Quantidade mensalidades 1° nível**	12 mês(es)
**Beneficiário**	Matheus Lotto de Almeida Souza
**Data Início**	15/06/2020
**Duração**	
**Moeda**	R$
**Valor Total das Mensalidades**	0,00 reais
**Justificativa**	
