# Supplementary material for: Psychophysiological Reactions of Internet Users Exposed to Fluoride Information and Disinformation: Protocol for a Randomized Controlled Trial
Source: JMIR Res Protoc. 2022 Jun 16;11(6):e39133. doi: 10.2196/39133 (PMC9247811; doi:10.2196/39133)
Supplement: Multimedia Appendix 2 [file resprot_v11i6e39133_app2.pdf]

VISUALIZAÇÃO DE DESPACHO

**Processo**	2019/27242-0
**Linha de Fomento**	Programas Regulares / Bolsas / No País / Doutorado - Fluxo Contínuo
**Situação**	Em Execução
**Vigência**	01/01/2021 a 30/04/2024
**Beneficiário**	Matheus Lotto de Almeida Souza
**Responsável**	Thiago Cruvinel da Silva
**Vínculo Institucional do Processo**	Faculdade de Odontologia de Bauru/FOB/USP
**Título**	Fake news em saúde: validação de um modelo de redes neurais para detecção de desinformação em Odontopediatria por meio de reações psicofisiológicas de usuários da Internet

Folha de Despacho para Reconsideração 001 - Doutorado

Resultado

Concedido

Datas do Despacho

**Emitido em :**	01/12/2020

Orçamento Consolidado

**Benefícios**	**Solicitado**		**Despacho**	
	**Valor (R$)**	**Valor (US$)**	**Valor (R$)**	**Valor (US$)**
Custeio				
Auxílio Instalação	0,00	0,00	0,00	0,00
Despesas de Transporte	0,00	0,00	0,00	0,00
Reserva Técnica de Bolsa	44.375,58	0,00	29.583,72	0,00
Total	44.375,58	0,00	29.583,72	0,00
Bolsas				
Doutorado	147.918,60	0,00	147.918,60	0,00
Total	147.918,60	0,00	147.918,60	0,00
Total Geral	192.294,18	0,00	177.502,32	0,00

Dados de Execução

**Data Início**	01/11/2020
**Duração**	42 mês(es)
**Data Término**	30/04/2024
**Área de alocação de recursos**	Saúde
**Relatório Científico (Quantidade)**	4
**Relatório Científico (Datas)**	10/10/2021<br>10/10/2022<br>10/10/2023<br>10/05/2024
**Ata de Defesa (Quantidade)**	1
**Ata de Defesa (Datas)**	15/11/2024
**Prestação de Contas (Quantidade)**	4

**Prestação de Contas (Datas)**	10/10/2021<br>10/10/2022<br>10/10/2023<br>10/05/2024
**Permite prorrogação da Bolsa?**	Sim

Observações / Transcrições / Frases

Observações ao Beneficiário

Comunicamos que sua solicitação de bolsa, constante do processo acima referido, foi analisada e aprovada pela FAPESP.

Por favor, aguarde o email com as instruções para confirmação de interesse pela concessão.

Esclarecemos que o tempo de bolsa já usufruído no doutorado pelo (a) bolsista será considerado pela FAPESP na duração máxima da bolsa.

Para conhecimento do conteúdo do despacho, por favor, acesse o Sistema SAGe (www.fapesp.br/sage), selecionando o item do menu Meus Processos>>Número do Processo e, em Mais Informações, a opção Despacho.

Com exceção de bolsa de Pós Doutorado, a transcrição do parecer está disponível exclusivamente para o orientador, sendo de sua responsabilidade escolher os trechos a serem compartilhados com o candidato.

Para qualquer consulta ou comunicação sobre esta correspondência, por favor, use exclusivamente os serviços do "Converse com a FAPESP" em www.fapesp.br/converse.

Atenciosamente,

Luiz Eugênio A. M. Mello
Diretor Científico

PENDÊNCIAS

- Comprovante de matrícula no doutorado
- Comprovante de conclusão do mestrado
- Comprovante de vigência (data de início e término) da bolsa doutorado CAPES

Frases para o Beneficiário

Não há frases associadas.

Transcrição de Parecer para o Beneficiário

Não há transcrição associada.

Frases para Termo de Outorga

Não há frases associadas.

Orçamento Detalhado - Quadros Resumos

Reserva Técnica de Bolsa - Solicitado

**Percentual para Reserva Técnica (País)**	30,00 %
**Percentual para Reserva Técnica (Exterior)**	0,00 %
**Dólar FAPESP**	5,55
**Valor da Reserva Técnica (R$)**	44.375,58
**Valor da Reserva Técnica (US$)**	0,00

Reserva Técnica de Bolsa - Despacho

**Percentual para Reserva Técnica (País)**	20,00 %
**Percentual para Reserva Técnica (Exterior)**	0,00 %
**Dólar FAPESP**	5,50
**Valor da Reserva Técnica (R$)**	29.583,72

**Valor da Reserva Técnica (US$)**	0,00

Bolsa

**Beneficiário**	Matheus Lotto de Almeida Souza	
**Modalidade**	Solicitado<br>Doutorado - DR-1	Despacho<br>Doutorado - DR-1
**Duração**	42 mês(es)	42 mês(es)
**Data Início**	05/09/2020	01/11/2020
**Moeda**	R$	R$
**Valor Unitário**	3.010,80	3.010,80
**Valor Total das Mensalidades**	147.918,60 reais	147.918,60 reais
**Justificativa**		

Bolsas do Beneficiário

Nenhuma bolsa encontrada.

Orçamento Detalhado - Itens de despesa

Reserva Técnica de Bolsa

**Percentual para Reserva Técnica (País)**	20,00 %
**Percentual para Reserva Técnica (Exterior)**	0,00 %
**Dólar FAPESP**	5,50
**Valor da Reserva Técnica (R$)**	29.583,72
**Valor da Reserva Técnica (US$)**	0,00

Bolsa

**Modalidade**	Doutorado - DR-1
**Quantidade mensalidades 1° nível**	12 mês(es)
**Beneficiário**	Matheus Lotto de Almeida Souza
**Data Início**	01/11/2020
**Duração**	42 mês(es)
**Moeda**	R$
**Valor Total das Mensalidades**	147.918,60 reais
**Justificativa**	
